# Scope+: an open source generalizable architecture for single-cell RNA-seq atlases at sample and cell levels

**DOI:** 10.1093/bioinformatics/btae727

**Published:** 2024-12-20

**Authors:** Danqing Yin, Yue Cao, Junyi Chen, Candice L Y Mak, Ken H O Yu, Jiaxuan Zhang, Jia Li, Yingxin Lin, Joshua W K Ho, Jean Y H Yang

**Affiliations:** Laboratory of Data Discovery for Health Limited (D24H), Pak Shek Kok, Hong Kong SAR, 999077, China; School of Biomedical Sciences, Li Ka Shing Faculty of Medicine, The University of Hong Kong, Pokfulam, Hong Kong SAR, 999077, China; Laboratory of Data Discovery for Health Limited (D24H), Pak Shek Kok, Hong Kong SAR, 999077, China; Charles Perkins Centre, University of Sydney, Camperdown, NSW, 2006, Australia; School of Mathematics and Statistics, University of Sydney, Camperdown, NSW, 2006, Australia; Sydney Precision Data Science Centre, University of Sydney, Camperdown, NSW, 2006, Australia; Laboratory of Data Discovery for Health Limited (D24H), Pak Shek Kok, Hong Kong SAR, 999077, China; School of Biomedical Sciences, Li Ka Shing Faculty of Medicine, The University of Hong Kong, Pokfulam, Hong Kong SAR, 999077, China; Laboratory of Data Discovery for Health Limited (D24H), Pak Shek Kok, Hong Kong SAR, 999077, China; Laboratory of Data Discovery for Health Limited (D24H), Pak Shek Kok, Hong Kong SAR, 999077, China; School of Biomedical Sciences, Li Ka Shing Faculty of Medicine, The University of Hong Kong, Pokfulam, Hong Kong SAR, 999077, China; Guangzhou National Laboratory, Guangzhou International Bio Island, Guangzhou, Guangdong Province 510005, China; Guangzhou National Laboratory, Guangzhou International Bio Island, Guangzhou, Guangdong Province 510005, China; State Key Laboratory of Respiratory Disease, National Clinical Research Center for Respiratory Disease, Guangzhou Institute of Respiratory Health, The First Affiliated Hospital of Guangzhou Medical University, Guangzhou, Guangdong Province, 510005, China; Laboratory of Data Discovery for Health Limited (D24H), Pak Shek Kok, Hong Kong SAR, 999077, China; Charles Perkins Centre, University of Sydney, Camperdown, NSW, 2006, Australia; School of Mathematics and Statistics, University of Sydney, Camperdown, NSW, 2006, Australia; Sydney Precision Data Science Centre, University of Sydney, Camperdown, NSW, 2006, Australia; Laboratory of Data Discovery for Health Limited (D24H), Pak Shek Kok, Hong Kong SAR, 999077, China; School of Biomedical Sciences, Li Ka Shing Faculty of Medicine, The University of Hong Kong, Pokfulam, Hong Kong SAR, 999077, China; Laboratory of Data Discovery for Health Limited (D24H), Pak Shek Kok, Hong Kong SAR, 999077, China; Charles Perkins Centre, University of Sydney, Camperdown, NSW, 2006, Australia; School of Mathematics and Statistics, University of Sydney, Camperdown, NSW, 2006, Australia; Sydney Precision Data Science Centre, University of Sydney, Camperdown, NSW, 2006, Australia

## Abstract

**Summary:**

With the recent advancement in single-cell RNA-sequencing technologies and the increased availability of integrative tools, challenges arise in easy and fast access to large collections of cell atlas. Existing cell atlas portals rarely are open sourced and adaptable, and do not support meta-analysis at cell level. Here, we present an open source, highly optimized and scalable architecture, named Scope+, to allow quick access, meta-analysis and cell-level selection of the atlas data. We applied this architecture to our well-curated 5 million COVID-19 blood and immune cells, as a portal called Covidscope. We achieved efficient access to atlas-scale data via three strategies, such as cell-as-unit data modelling, novel database optimization techniques and innovative software architectural design. Scope+ serves as an open source architecture for researchers to build on with their own atlas.

**Availability and implementation:**

The COVID-19 web portal, data and meta-analysis are available on Covidscope (https://covidsc.d24h.hk/). User tutorials on how to implement Scope+ architecture with their atlases can be found at https://hiyin.github.io/scopeplus-user-tutorial/. Scope+ source code can be found at https://doi.org/10.5281/zenodo.14174632 and https://github.com/hiyin/scopeplus.

## 1 Introduction

Single-cell technologies have become one of the most powerful techniques for understanding cell identity and function at the individual cell level. Despite having a myriad of single-cell RNA-sequencing (scRNA-seq) datasets from large cohort (Lin *et al.* 2022, Jin *et al.* 2021), it remains a challenge to effectively access and analyse multiple datasets jointly. A fundamental challenge is due to the characteristics of scRNA-seq data, where the number of genes (i.e. rows) is typically over 20 000 and the number of cells (i.e. columns) is in the range of hundreds of thousands. Traditional relational databases are incapable of storing data at the atlas level of magnitude. Recently, efforts resulted in the creation of multiple atlases such as the Human Cell Atlas (Regev *et al.* 2017, Roy and Conroy 2018, Rozenblatt-Rosen *et al.* 2020, Nieto *et al.* 2021, Cervia *et al.* 2022, Li *et al.* 2022, Zeng *et al.* 2022). However, most existing scRNA-seq atlas portals are not open sourced, and are not released as a generalizable architecture for adaptation. Additionally, they are unable to subset the data at the unit of cell for selective downstream analysis (Supplementary Table S1).

Here, we present Scope+, an open source, transferable and scalable software architecture for cell atlas portal that facilitates effective meta-analysis downstream analysis for scRNA-seq data. Compared to over 20 existing scRNA-seq atlases, Scope+ presents the first generalizable software architecture for scRNA-seq data. Scope+ architecture is realized in Covidscope web portal where we gathered approximately 5 million single PBMC cells from 20 COVID-19 studies globally as a valuable community resource. Scope+ addresses the high-throughput data challenges associated with dynamic subsetting and complex querying of 12.7B matrix count and ∼5M meta information with three key innovations. These are (i) database optimization techniques to allow extremely fast (10–6000x faster) query of the 12.7 B matrix based on the 5M meta data; (ii) ultrafast pagination and cell-as-unit data model enables selection of specific cells in meta data across integrated datasets for comparison; and (iii) novel architecture design to support large-scale bioinformatics data filtering, and visualization tasks in the web portal.

## 2 Scope+ implementation

### 2.1 Scope+: an open source innovative software architecture for large-scale data quick access and meta-analysis at cellular resolution

**Figure 1.**
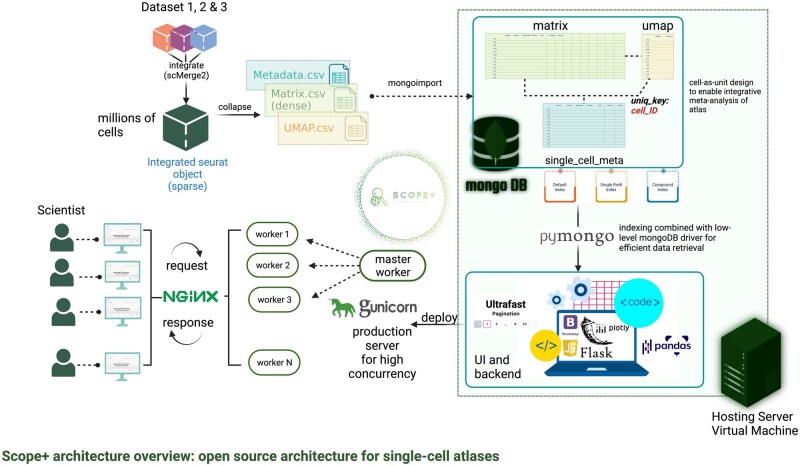
Scope+ architecture diagram behind Covidscope web portal (up: architecture abstract layers; bottom: architecture technical overview). The Scope+ architecture behind Covidscope is divided into five layers, i.e. presentation layer, interaction layer, service layer, application logic layer, and data storage layer. Each layer is composed of one to several web application development tools with their functional description.

Scope+ is a five-layered web application architecture (Fig. 1), with presentation layer, application logic layer and data storage layer being the three primary layers. MongoDB is applied for large scRNA-Seq data persistence in the database storage layer and Flask is applied in the application logic layer to store web logics (see Supplementary Methods). Four main functions of the portal are: fast cell sorting, interactive visualization on cell level, visualization on sample level, and data download. Cell sorting is an interactive table which provides fast and intuitive cell-level search with metadata on the scRNA-seq data (Supplementary Fig. S1a). Users can filter cells interested by various attributes such as sample, age, health status, and cell types. Interactive visualization at cell level includes UMAP (see Supplementary Methods) plots of the selected cells coloured by the metadata (Supplementary Fig. S1b), and gene expression across cell types (Supplementary Fig. S1c). We also include sample level feature visualization including cell type proportions (Supplementary Fig. S1d), marker gene expressions, and pathway scores to provide comprehensive knowledge of the sample level information (Supplementary Fig. S2). Users can download all the queried cell results with the 10x genomics format along with the metadata and the feature level data from the website.

Thanks to our optimization of the database using MongoDB indexing mechanism in Scope+, the performance of the cell sorting of the website is fast. We tested the performance of cell sorting by metadata and gene search in the UMAP plot across our optimized indexing and pagination strategies (Supplementary Fig. S1e and f). The website queries cells by selecting condition through cell sorting within 0.06 milliseconds; it can also search gene expression for selected cells from the large cell matrix (∼5 million cells and ∼30 000 genes) within 6.7 s. It is by average >3 folds in cell sorting and >6000 folds faster than the baseline database. The result indicates that our noSQL based database can achieve fast query on scRNA-seq data.

Secondly, easy expression matrix accessibility through fast metadata subsetting is achieved by the modelling of the NoSQL database (Supplementary Fig. S3). The NoSQL database utilizes the inherent dropout of the scRNA-seq data, where only a small portion of the transcriptome is represented in each cell (Gong *et al.* 2018, Qiu 2020). It can index cells with cell identities featured by a series of sparse omics information and metadata as a key-values model. This solution reduces the data scale and enhances the data retrieval speed by indexing multiple keys. Particularly, we further enhance the performance of the website by three complementary optimizations including: (i) the choice of low level driver, (ii) the pagination mechanism, and (iii) the database indexing.

Our curated NoSQL database is primarily composed of four tables of (i) cell count matrix, (ii) metadata of the cells, donors or datasets, (iii) their computed UMAP coordinates and (iv) derived features for individual donors generated from the method scFeatures (Cao *et al.* 2022). The design of our web application is highly modular, scalable, and optimized for runtime responsiveness. Together, this enables us to provide a centralized portal supporting quick database query, visualization, data download, and integrative analysis of any scRNA-seq data.

### 2.2 Covidscope: an COVID-19 scRNA-seq portal

We applied Scope+ to our well-curated COVID-19 atlas collections of integrated data and released it in a web-based portal, Covidscope (Supplementary Fig. S4). Our integrated data collection consists of scRNA-seq data from 20 published COVID-19 single-cell studies (Supplementary Table S2) that consist of close to 5 million cells (4 899 035) in total from blood cells extracted from almost 1000 donors across 9 different countries. This portal is accessible at https://covidsc.d24h.hk.

To construct a COVID-19 ‘analytic-ready cell atlas’, we unified 20 columns of technical, biological and clinical metadata (Supplementary Table S3). Together these datasets include 1298 samples from 963 individuals, with diverse disease severity (15.2% Healthy, 40.8% Mild/Moderate, 32.8% Severe/Critical, 1.8% Asymptomatic); disease outcome (39.4% Discharged, 7.4% Deceased, 6.4% Hospitalized, 1.0% Not hospitalized); gender (41.8% Female and 49.7% Male, 8.5% Unknown) (Supplementary Fig. S5) and other characteristics.

Covidscope built using Scope+ demonstrates notable advancements compared to existing scRNA-seq atlases (Supplementary Data S1). One distinction is its ability to perform advanced query searches that select for specific cells of interest by patient metadata such as by age and gender, across all datasets. In comparison, many existing atlases only allow for the download of the entire dataset. Covidscope also offers patient-level analysis such as cell-type specific pathway enrichment and cell-cell communication information and their comparison between different severity conditions. In comparison, existing COVID-19 atlases such as SCovid only provide basic data exploration such as UMAP plot visualization coloured by cell types. In terms of web server performance, Covidscope ranked second out of the nine scRNA-seq atlases compared, as measured by Google Lighthouse.

## 3 Results

### 3.1 Case study 1: cell level analysis of a subset cohort analysis

Integrated data from multiple studies with carefully curated metadata architected under Scope+ enables many new possibilities for downstream analyses. For example, using condition outcomes and cell type labels, one could perform case–control studies as well as multi-conditional studies examining composition change, expression shift, perturbation analysis and a range of other analyses in a cell type specific manner.

To demonstrate the power of an integrated data atlas, we examined the existence of a common signature that distinguishes cells from mild and severe COVID-19 patients across multiple datasets. We focused on CD14 monocytes as this is a key player in COVID-19. Scope+ allows us to selectively retrieve all relevant data corresponding to the CD14 monocyte cells. Using the Leiden community detection algorithm in R, we obtained 23 subclusters with multiple clusters enriched in severe cells (Supplementary Fig. S6a). In these clusters, the proportion of cells from severe COVID-19 patients reached over 75%, marginally higher than the overall proportion of ‘severe cells’ in the CD14 monocytes population (53%), suggesting a potential ‘severe’ signature. Cluster 18 potentially harboured common severe signatures across multiple cohorts, as it contained a significant number of cells (over 300) across seven datasets (Supplementary Fig. S7a) and had a high dataset diversity, as demonstrated by the high Shannon diversity index (Supplementary Fig. S6b). Differentially expressed gene analysis in cluster 18 (Supplementary Fig. S6c) revealed that eight out of the top 10 up-regulated genes belonged to the immunoglobulin gene family (Supplementary Fig. S7b). Multiple studies in literature consistently point to increased immunoglobulin antibodies level in severe patients compared to mild and healthy controls (Luo *et al.* 2021, Cervia *et al.* 2022, Välikangas *et al.* 2022). Together, we demonstrate the data atlas promotes the identification of common severe signatures across multiple cohorts.

### 3.2 Case study 2: sample level analysis through integrative analysis of multiple datasets

One of the advantages of the Scope+ is that it enables the integrative analysis of multiple datasets, which opens the opportunity to examine various sub-populations across many datasets. One such question is the molecular difference underlying mild and severe outcomes in a given age group, which cannot be easily addressed without combining multiple datasets. For example, while ten COVID-19 studies included in our atlas surveyed middle-aged patients of 41–50, the majority of these studies contain fewer than 10 mild patients (Supplementary Fig. S8). Similar observations can be found in all other age groups, which pose a significant challenge for any downstream analysis. In contrast, by combining all 20 datasets, Scope+ enables us to selectively examine 128 middle-aged patients of 41–50 and 131 elderly patients of 71–80 and to derive biological discovery between mild and severe conditions in these two age groups (Supplementary Fig. S9a).

To explore the differences between mild and severe patients, we used scFeatures to generate the molecular representation of each individual using a set of feature types. This was then followed by classification of the mild and severe conditions using each of the feature types. Majority of the feature types resulted in different prediction accuracy between the 71 and 80 age group compared to the 41 and 50 (Supplementary Fig. S9b). For example, the feature type ‘pathway GSVA comorbidity’, which includes the pathway scores of several well-known comorbidities associated with COVID-19 outcomes (Williamson *et al.* 2020) achieved an accuracy of over 0.8 for 71–80 but only over 0.65 for 41–50. Further examination revealed that the severe patients in the 71–80 age group had noticeably higher comorbidity pathway scores compared to the mild patients (Supplementary Fig. S9c). This pattern was not observed in the 41–50 age group.

For the 41–50 age group, the top performing four feature types were all related to gene expression (i.e. ‘gene mean celltype’, ‘gene proportion celltype’, ‘gene mean aggregated’ and ‘gene proportion aggregated’), achieving over 0.75 accuracy. Further analysis of top 20 features from the feature type ‘gene mean celltype’ revealed the cell type CD4 T, out of the total of 15 cell types, appeared 7 times out of the 20 features. This over-representation potentially suggests the importance of CD4 T cells in COVID-19 severity. Thus, through integrative analysis of patients across multiple datasets, we highlight the differing roles of certain feature types in affecting COVID-19 outcomes in different age groups and reveal biological insights into the mild and severe conditions.

## 4 Discussion

In this paper, we propose an architecture Scope+ that enables fast access, query, and analysis of scRNA-seq atlas at the scale of millions of cells. Using the architecture, we hosted a portal, Covidscope, containing almost 5 million PBMC from COVID-19 patients. The Covidscope portal provides users access at an unparalleled speed to COVID-19 gene expression data for cell clustering visualization and flexible downstream meta-analysis by providing multi-conditional filtering. The collection of five million COVID-19 cells offers a valuable resource to the single-cell community to uncover critical insights. For example, researchers can use the integrated data to more accurately identify novel cell types and cell subpopulations with the latest scalable models (Cheng *et al.* 2023, Zheng *et al.* 2024), as datasets’ batch effect has been minimized. This atlas, where the patient metadata across 20 studies has been carefully standardized through manual effort, also serves as a source of training data for the latest single-cell foundation models (Cui *et al.* 2024, Hao *et al.* 2024). This unlocks the power of these foundation models to identify cellular changes in COVID-19 disease and guide drug development.

The Scope+ architecture is the first generalizable atlas architecture made open source, with numerous efforts in summarizing and characterizing features from previous atlas portals. Scope+ is of great extensibility, by open-sourcing our web portal and providing an implementation tutorial (https://hiyin.github.io/scopeplus-user-tutorial/), we invite researchers to extend our web portal to address their specific questions. We have made the web portal generalizable to support the hosting and exploration of any scRNA-seq data.

## Supplementary Material

btae727_Supplementary_Data

## Data Availability

The COVID-19 web portal, data and meta-analysis are available on Covidscope (https://covidsc.d24h.hk/). User tutorials on how to implement Scope+ architecture with their atlas-es can be found at https://hiyin.github.io/scopeplus-user-tutorial/. Scope+ source code can be found at https://doi.org/10.5281/zenodo.14174632 and https://github.com/hiyin/scopeplus.
