# Supplementary material for: Scope+: an open source generalizable architecture for single-cell RNA-seq atlases at sample and cell levels
Source: Bioinformatics. 2024 Dec 20;41(1):btae727. doi: 10.1093/bioinformatics/btae727 (PMC11755096; doi:10.1093/bioinformatics/btae727)
Supplement: btae727_Supplementary_Data [file btae727_supplementary_data.zip › MS-Covid-19 on the cloud - revision - supp file.docx]

### **Supplementary File**

###

### **Supplementary Methods**

###

### ***Data Description***

A collection of almost 5 million blood and immune cells extracted from almost 1,000 COVID-19 patients across 20 studies worldwide was used in this study. Supplementary Table 2 provides a summary of these 20 data sets, including the data accession ID. Each data set was transformed into a gene-by-cell raw count expression matrix. We performed size factor standardisation and log transformation matrix using the logNormCount function in the R package scater (version 1.16.2) and generated log-transformed gene expression matrices for downstream analysis. We performed cell type classification and re-annotated each data set with scClassify [(Y. Lin et al. 2020)](https://paperpile.com/c/oLcOhy/yzKXA) for all data regardless of the original annotations. The data collection was merged and harmonised using scMerge2 [(Y. Lin et al. 2023)](https://paperpile.com/c/oLcOhy/INMRF) in R software and stored in RDS file format.

### ***Data preprocessing***

UMAP coordinates

UMAP coordinates were generated based on the top 50 principal components of the scMerge2 integrated matrix. The function *umap* implemented in the R package uwot was used, with *min_dist = 0.3*.

Patient based interpretable features

We applied scFeatures [(Y. Cao et al. 2022)](https://paperpile.com/c/oLcOhy/BByOM), a single-cell data feature extraction tool that we previously developed to extract a range of interpretable features. The features span across multiple categories, such as cell type proportion, cell type specific gene expression and cell type specific pathway enrichment and form the molecular representation of individuals. This provides molecular representations for all patients in the 20 COVID-19 studies, which are then visualised as interactive plots on the web portal.

Extract-transform-load (ETL) process

To process files for data import, Python scripts were used for converting RDS in section (i) to a CSV (comma-separated value) format, in which each line of the CSV file represents a record. The Unix command-line was used to import CSV to MongoDB using the “mongoimport” API. The imported data in the database follows the same structure as the original CSV, with a header as their column names. Data preprocessing scripts for the above mentioned processes is available on <https://github.com/hiyin/scopeplus-scripts>.

#### ***Software architecture and implementation***

The architecture Scope+ consists of three principal layers.

(i) Data storage layer

We designed separate collections for cell, cell type, and sample level features. Cell level features including the expression matrix, UMAP coordinates, and the cell type level feature as the metadata are stored in the **cov19atlas** database. The molecular features generated from scFeatures (see iv) including raw proportion, gene expression proportion and the pathway means score for each combination of sample and cell type are stored in the **scFeatures** database. More details can be found in Supplementary Fig. 3. The molecular features generated from scFeatures (see iv) are stored into the **scFeatures** database. Single-cell meta information is stored into the **cov19atlas** database, with three collections namely single_cell_meta, umap and matrix, a universal id is created to cross-link these collections.

(ii) Presentation layer

HTML5 and Bootstrap (getboostrap.com) were used to build the modern responsive web user interface. JavaScript (www.javascript.com) and jQuery library (jquery.com) were used to manage dynamic content alteration, event, animations and asynchronous data transfer.

(iii) Application logic layer

A collection of APIs built on PyMongo, DataTables (<https://datatables.net>) and Flask make up the application’s logic. The *PyMongo API* handles the database connection and information retrieval. The self-constructed *search API* performs translation of search conditions into database query. The *DataTables API* displays paginated search results from MongoDB into tables using server-side rendering.

Data visualisation API for cell and individual level information

All diagnostic plots such as scatterplots, box plots, and bar charts presented on the website were implemented using the Plotly python package [(Inc. 2015)](https://paperpile.com/c/oLcOhy/mvoRp). On the cell level, we implemented UMAP plots to visualise the dimension-reduced cell expression using the *plotly.express.scatter* function. The box plot for gene expression levels across cell types was implemented using the *plotly.express.box* function.

We implemented several types of figures to demonstrate the individual level feature representation. The composition plot to visualise the cell type composition of patients was generated by the *plotly.express.bar* function. The visualisation of the distribution of individual feature values was implemented in several functions including box plot and heatmap. The box plot of pathway enrichment score across cell types was generated by the *plotly.express.box* function. The heatmap with dendrogram of mean gene expression and pathway enrichment score across cell types was generated by the *dash_bio* python package [(Hossain, Calloway, and Lippa 2019)](https://paperpile.com/c/oLcOhy/atPMD) using the function *dash_bio.Clustergram.*

#### ***Optimisation***

The Scope+ architecture implements three key optimisation strategies through:

1) Choice of driver: a low-level database driver PyMongo is used to interface the application with MongoDB. MongoDB cursor is returned directly from database query without converting back to Python object.

2) Pagination mechanism: MongoDB’s default ObjectId index is used for pagination of search results, server-side processing is enabled by setting “serverSide: true” in *DataTable API*.

3) Database indexing: frequently searched fields in MongoDB collections are indexed using *MongoDB API* (*db.matrix.createIndex({column_name:1}))*.

####

#### ***Statistical analysis***

Meta-analysis of CD14 monocyte cell populations

To demonstrate the power of using the atlas-scale database to reveal insights into COVID-19, we focused on the CD14 monocytes, which is one of the key cell types in COVID-19 response. Using *Monocle3* [(J. Cao et al. 2019)](https://paperpile.com/c/oLcOhy/k75gA), which is found to be the best scRNA-seq clustering method for estimating the number of cell types [(L. Yu et al. 2022)](https://paperpile.com/c/oLcOhy/k0n6M), we clustered the CD14 monocytes into subpopulations. In order to prioritise clusters for downstream analysis, we used two criteria of (1) the proportion of severe cells in the cluster and (2) the diversity of the cluster in terms of the number of cells from each study and the number of studies that appeared in each cluster. We quantified criteria 2 using Shannon diversity index. Using these two criteria, we identified cluster 18 as a particularly interesting cluster as it was enriched in severe cells and had a high diversity index, containing a significant number of cells from seven studies. The standard DE analysis was then performed using scran to examine the up-regulated genes in cluster 18 compared to the remaining clusters.

Meta-analysis for individuals between 41-50 age group

To explore whether the mechanism underlying disease severity is the same across patients of different age groups, we selected the mild/moderate (referred to as mild) and severe/critical patients (referred to as severe) from two age groups of 41-50 and 71-80. We included all patients across all data sets in the atlas in order to obtain sufficient sample sizes. scFeatures was used to generate multiple feature types for each individual. To construct the comorbidity pathway features, we queried the Disease Ontology database [(G. Yu et al. 2015)](https://paperpile.com/c/oLcOhy/1JuGV) using the following terms of “heart”, “cardiovascular”, “hypertension”, and “diabetes” to obtain the relevant pathways and genes. Using the feature representations of individuals and the severity outcome, the prediction performance of each feature type was obtained using a linear kernel SVMwith three-fold cross-validation, repeated 20 times. As the outcome class has an imbalanced sample size, balanced accuracy was used as the evaluation metric.

###

###

### **Supplementary Tables**

**Supplementary Table 1: Examples of published atlases that collate single-cell omics data from multiple studies and comparison to Scope+**

| **Name** | **Publication** | **Web portal** | **Sample metadata / Analytical results / Both** | **Type of samples (human)** | **Number of single cells* (million)** | **Open source** | **Generalizable architecture with source code** | **Integrative analysis at cell level** |
| --- | --- | --- | --- | --- | --- | --- | --- | --- |
| Scope+ | This study | https://covidsc.d24h.hk | Both | Healthy and diseased | 4.8 | Yes | Yes | Yes |
| Human Cell Atlas | [(Regev et al. 2017)](https://paperpile.com/c/oLcOhy/zIDr) | https://data.humancellatlas.org/ | Sample metadata | Healthy and diseased | 62.1 | No | No | No |
| hECA | [(S. Chen et al. 2022)](https://paperpile.com/c/oLcOhy/qUTN) | http://eca.xglab.tech/ | Both | Healthy | 1.09 | No | No | Yes |
| Tabula Sapiens Consortium | [(Tabula Sapiens Consortium* et al. 2022)](https://paperpile.com/c/oLcOhy/KHsa) | https://tabula-sapiens-portal.ds.czbiohub.org/ | Sample metadata | Healthy | 0.5 | No | No | No |
| HuBMAP | [(HuBMAP Consortium 2019)](https://paperpile.com/c/oLcOhy/4J3I) | https://portal.hubmapconsortium.org/ | Sample metadata | Healthy | Not stated | Yes | Only web portal UI | No |
| Human Tumor Atlas Network | [(Rozenblatt-Rosen et al. 2020)](https://paperpile.com/c/oLcOhy/YttH) | https://humantumoratlas.org/ | Sample metadata | Cancer | Not stated | No | No | No |
| Tumor Immune Cell Atlas | [(Nieto et al. 2021)](https://paperpile.com/c/oLcOhy/EJWR) | https://singlecellgenomics-cnag-crg.shinyapps.io/TICA/ | Both | Cancer | 0.3 | Yes, shiny app | Only web portal UI | No |
| Cancer Single-cell Expression Map | [(Zeng et al. 2022)](https://paperpile.com/c/oLcOhy/DNnW) | https://ngdc.cncb.ac.cn/cancerscem/index | Both | Cancer | 6.8 | No | No | No |
| Single-Cell Immunology Of SARS-CoV-2 Infection | [(Tian et al. 2022)](https://paperpile.com/c/oLcOhy/uDto) | https://atlas.fredhutch.org/fredhutch/covid/ | Sample metadata | COVID-19 | 3.2 | No | No | No |
| COVID-19 Cell Atlas | [(Sungnak et al. 2020)](https://paperpile.com/c/oLcOhy/Uiz1) | www.covid19cellatlas.org | Sample metadata | Healthy and COVID-19 | Not stated | No | No | No |
| SCovid | [(Qi et al. 2022)](https://paperpile.com/c/oLcOhy/h14r) | http://bio-annotation.cn/scovid/#/ | Both | COVID-19 | 3.5 | No | No | No |
| ToppCell | [(Jin et al. 2021)](https://paperpile.com/c/oLcOhy/RqZi) | http://toppcell.cchmc.org/ | Analytical results | COVID-19, cancer, and healthy | 0.48 (COVID-19) | No | No | No |
| DISCO | [(Li et al. 2022)](https://paperpile.com/c/oLcOhy/vEKG) | https://www.immunesinglecell.org/ | Both | 15 tissues, COVID-19 | 2.6 (COVID-19) | No | No | No |
| TIGER | [(Z. Chen et al. 2023)](https://paperpile.com/c/oLcOhy/XqxS) | http://tiger.canceromics.org/#/ | Both | Cancer | 2.1 | No | No | No |
| ABC | [(Gao et al. 2023)](https://paperpile.com/c/oLcOhy/unck) | http://abc.sklehabc.com | Both | Blood disease | Not stated | No | No | No |
| PlaqView | [(Ma et al. 2022)](https://paperpile.com/c/oLcOhy/r7q6) | https://www.plaqview.com/ | Both | Cardiovascular disease | 2.4 | Yes, shiny app | Only web portal UI | No |
| SPICA | [(Andreatta et al. 2022)](https://paperpile.com/c/oLcOhy/WAng) | https://spica.unil.ch | Both | Healthy and disease | Not stated | No | No | No |
| TCAC | [(Zhou et al. 2022)](https://paperpile.com/c/oLcOhy/6KZ7) | https://taca.lerner.ccf.org/ | Both | Alzhemier’s disease | 1.1 | No | No | No |
| CellDepot | [(D. Lin et al. 2021)](https://paperpile.com/c/oLcOhy/G7hv) | http://celldepot.bxgenomics.com | Both | Healthy and diseased | Not stated | Yes | No | No |
| scAPAatlas | [(Yang et al. 2022)](https://paperpile.com/c/oLcOhy/wltW) | http://www.bioailab.com:3838/scAPAatlas/ | Both | Healthy tissues | 0.8 | No | No | No |
| IAAA | [(Shen et al. 2022)](https://paperpile.com/c/oLcOhy/6li8) | http://galaxy.ustc.edu.cn/IAAA | Both | Autoimmune disease | 0.8 | No | No | No |
| CZ CELLxGENE Discover | [(CZI Single-Cell Biology Program et al. 2023)](https://paperpile.com/c/oLcOhy/v6xZ) | https://cellxgene.cziscience.com/ | Both | Both | 90.1 | Yes | No | No |
| scIBD | [(Nie et al. 2023)](https://paperpile.com/c/oLcOhy/kd0g) | http://scibd.cn/ | Both | Inflammatory bowel disease | 1.14 | No | No | No |
| Single Cell Atlas | [(Pan et al. 2024)](https://paperpile.com/c/oLcOhy/iZVz) | https://www.singlecellatlas.org/ | Both | Healthy human | 200.2 | No | No | No |
| Brain Cell atlas | [(X. Chen et al. 2024)](https://paperpile.com/c/oLcOhy/M3kb) | https://www.braincellatlas.org/index | Both | Brain | 26.3 | No | No | No |

*Number of single cells collated by the resource at the time of writing, September 2024

**Supplementary Table 2: Overview of the 20 COVID-19 PBMC data sets used in this study.**

| **Data set** | **Data accession ID** | **Reference** | **Number of cells** | **Number of patients** |
| --- | --- | --- | --- | --- |
| Arunachalam et al. | GSE155673 | [(Arunachalam et al. 2020)](https://paperpile.com/c/oLcOhy/jVU8m) | 56,639 | 12 |
| Bost et al. | GSE157344 | [(Bost et al. 2021)](https://paperpile.com/c/oLcOhy/jUu37) | 50,284 | 33 |
| Combat et al. | EGAS00001005493 | [(COvid-19 Multi-omics Blood ATlas (COMBAT) Consortium et al. 2021)](https://paperpile.com/c/oLcOhy/a4mMe) | 783,704 | 140 |
| Combes et al. | GSE163668 | [(Combes et al. 2021)](https://paperpile.com/c/oLcOhy/W9Ogo) | 111,990 | 44 |
| Lee et al. | GSE147507 | [(Lee et al. 2020)](https://paperpile.com/c/oLcOhy/eoSuj) | 59,572 | 17 |
| Liu et al. | GSE161918 | [(Liu et al. 2021)](https://paperpile.com/c/oLcOhy/qHC88) | 411,902 | 47 |
| Ramaswamy et al. | GSE166489 | [(Ramaswamy et al. 2021)](https://paperpile.com/c/oLcOhy/Ms85l) | 271,267 | 32 |
| Ren et al. | GSE158055 | [(Ren et al. 2021)](https://paperpile.com/c/oLcOhy/bg3mM) | 999,462 | 151 |
| Schulte-schrepping et al. | EGAS00001004571 | [(Schulte-Schrepping et al. 2020)](https://paperpile.com/c/oLcOhy/ItQbY) | 328,780 | 74 |
| Schuurman et al. | GSE164948 | [(Schuurman et al. 2021)](https://paperpile.com/c/oLcOhy/Jqn7F) | 32,384 | 20 |
| Silvin et al. | E-MTAB-9221 | [(Silvin et al. 2020)](https://paperpile.com/c/oLcOhy/3HL9A) | 6,960 | 10 |
| Sinha et al. | GSE157789 | [(Sinha et al. 2022)](https://paperpile.com/c/oLcOhy/WPR3L) | 80,994 | 14 |
| Stephenson et al. | E-MTAB-10026 | [(Stephenson et al. 2021)](https://paperpile.com/c/oLcOhy/hv3Wh) | 643,071 | 130 |
| Su et al. | E-MTAB-9357 | [(Su et al. 2020)](https://paperpile.com/c/oLcOhy/6guox) | 538,210 | 143 |
| Thompson et al. | GSE166992 | [(Thompson et al. 2021)](https://paperpile.com/c/oLcOhy/4lqWX) | 63,895 | 8 |
| Unterman et al. | GSE155224 | [(Unterman et al. 2022)](https://paperpile.com/c/oLcOhy/DRwrW) | 80,789 | 10 |
| Wilk et al. | GSE174072 | [(Wilk et al. 2021)](https://paperpile.com/c/oLcOhy/ieply) | 174,753 | 39 |
| Yao et al. | GSE154567 | [(Yao et al. 2021)](https://paperpile.com/c/oLcOhy/xV0wA) | 69,983 | 17 |
| Zhao et al. | CNP0001250 (data available at https://figshare.com/articles/dataset/seu_obj_h5ad/16922467) | [(Zhao et al. 2021)](https://paperpile.com/c/oLcOhy/2ufUk) | 88,374 | 19 |
| Zhu et al. | CNP0001102 | [(Zhu et al. 2020)](https://paperpile.com/c/oLcOhy/0Cf9j) | 46,022 | 3 |
| Total |  |  | 4,899,035 | 963 |

**Supplementary Table3: Overview of the metadata as curated in the 20 COVID-19 data sets. For categorical value types, we report the categories.**

| **Metadata** | **Patient or study characteristic** | **Values for categorical variables** |
| --- | --- | --- |
| Dataset | Study |  |
| Tissue | Study | “PBMC” (Peripheral blood mononuclear cells)  “PFMC” (pleural effusion)  “Whole blood” |
| Sample_type | Study | “Frozen PBMC”  “Fresh PBMC“  “Whole blood“  “Fresh PFMC“ |
| Protocol | Study | “10X 3'“  “10X 5'“  "Rhapsody"  “Seq-Well“  “DNBelab C4“ |
| Technology | Study | “CITE-seq”  “scRNA-seq” |
| Sample_time | Patient | “Healthy“  “Progression“  “Convalescence“  “Others“  “Unknown“ |
| Sample_id | Patient |  |
| Patient_id | Patient |  |
| Disease | Patient | “Healthy“  “COVID-19“  “Influenza“  “Sepsis“  “MIS“ (multisystem inflammatory syndrome)  “CAP“ (community-acquired pneumonia)  “LPS “(Lipopolysaccharides)  “Non-COVID-19 severe respiratory illness“  “Others“ |
| Severity | Patient | “Healthy”  “Mild/Moderate”  “Severe/Critical”  “Asymptomatic”  “Convalescence”  “Non-covid”  “Unknown” |
| WHO_scores  (Categorical) | Patient | “0”  “1”  “1 or 2”  “2”  “3”  “4”  “5”  “6”  “7”  “NA” |
| Ethnicity | Patient | "Black"  "White"  "Asian"  "Hispanic/Latino"  "Others"  "Unknown" |
| Gender | Patient | “Female”  “Male”  “Unknown” |
| Age | Patient |  |
| Age_category | Patient | "<=18"  "18-30"  "31-40"  "41-50"  "51-60"  "61-70"  "71-80"  "80+"  "Unknown" |
| Days_from_onset_of_symptoms | Patient |  |
| Outcome | Patient | "Deceased"  "Discharged"  "Healthy"  "Hospitalized"  "Not hospitalized"  "Unknown" |
| BMI | Patient |  |
| PreExistingHypertension | Patient | “Yes”  “No”  “Unknown” |
| PreExistingHeartDisease | Patient | “Yes”  “No”  “Unknown” |

###

### **Supplementary Figures**


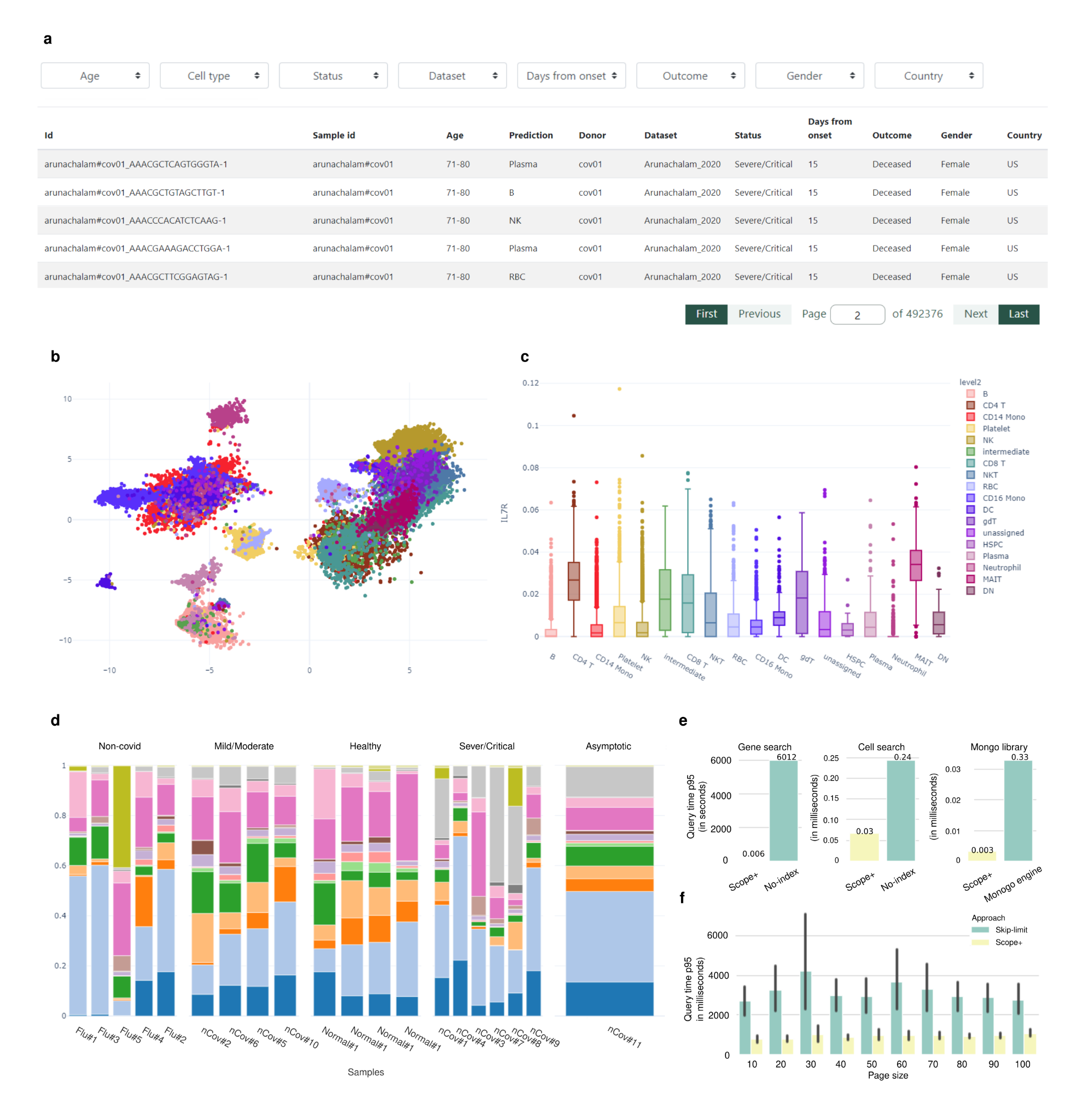


**Supplementary Figure 1:** **Covidscope web portal for COVID-19 single-cell meta analysis.**

**a** Demonstrates the interactive table for users to perform cell sorting through subsetting cells through metadata. Users can subset cells by attributes of interest. **b** Is the UMAP plot of cells from a specific data set (Lee et al, 2022) colored by the annotated cell types. **c** Shows the expression level of the selected gene across cell types. X-axis stands for the cell types while y-axis stands for the normalised expression level. **d** Is the cell type proportion across various disease severities of the Lee et al data set. Each bar represents one sample in the data set. Y-axis is the cell type proportion of each sample. **e** Demonstrates the time of different indexing test cases, each pair of the bar plots corresponds to gene expression searching, cell sorting, and library selection. **f** Demonstrates the time performance of pagination which x axis is the page size of the cell sorting table and y axis is the query time.


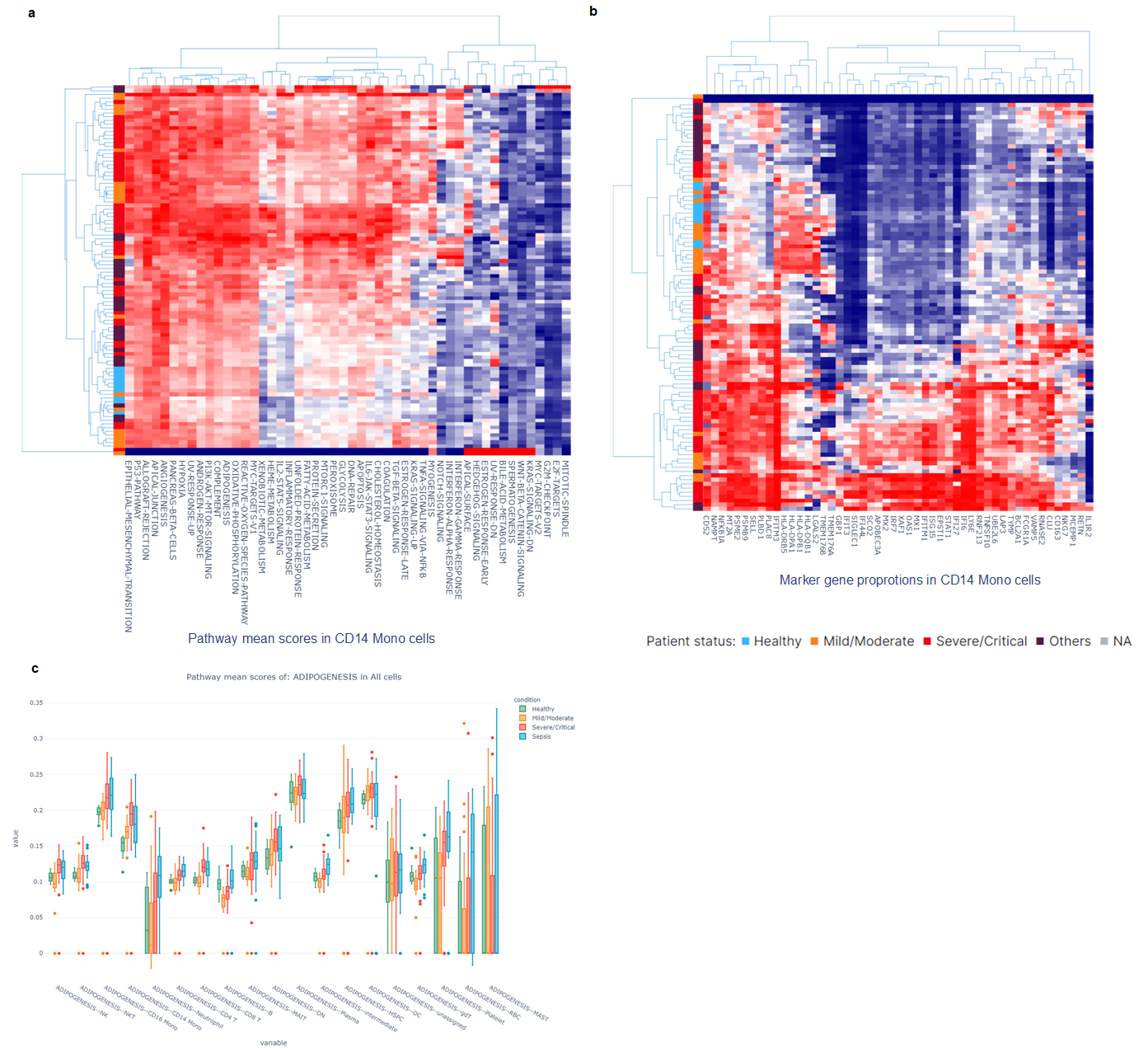


**Supplementary Figure 2. Screenshots for the sample level features on the websites.**

**a** Heatmap shows the pathway score, constructed on cell type specific manner. Each row represents a patient, with the sidebar colour annotated by their disease status and each column represents a pathway. Red colour indicates a higher score and blue colour indiates a lower score. **b** Heatmap shows the proportion of gene expression, constructed on cell type specific manner. Each row represents a patient, with the sidebar colour annotated by their disease status and each column represents a marker gene. Red colour indicates a higher score and blue colour indicates a lower score. **c** Boxplot shows the distribution of the pathway score in the patients, coloured by COVID-19 outcomes.


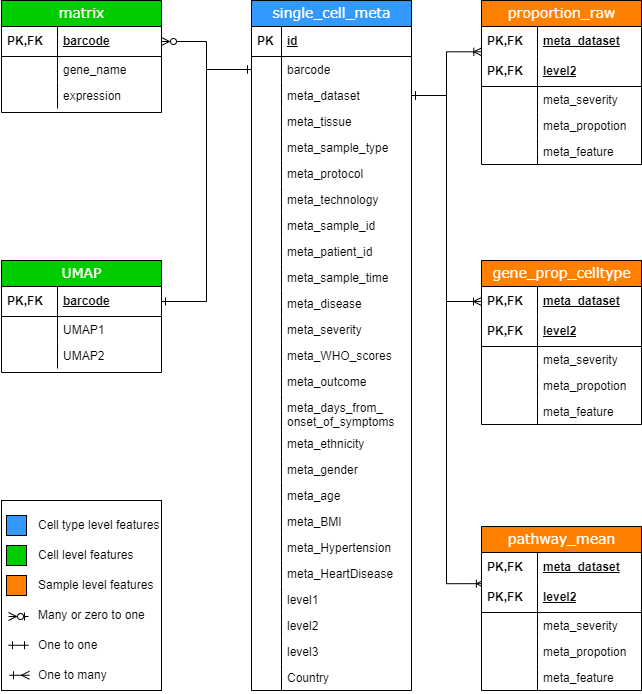


**Supplementary Figure 3. Database schema of the Covidscope data portal.**

Each rectangular box refers to an individual data collection in the MongoDB which Covidscope hosts. Primary keys (PK), foreign keys (FK), and other attributes within data collections are illustrated. Note that levels 1 ~3 refer to a different level of cell type annotation predicted by the scClassify. There are separate collections for cell, cell type, and sample level features. Cell level features include the expression matrix and the UMAP coordinates. The cell type level feature includes the metadata collection. Sample level features include the raw proportion, gene expression proportion and the pathway means score for each cell type. Cell and gene level collections are interconnected by cell barcodes. Sample level collections are connected by meta_dataset and the level2 cell type annotation.

**
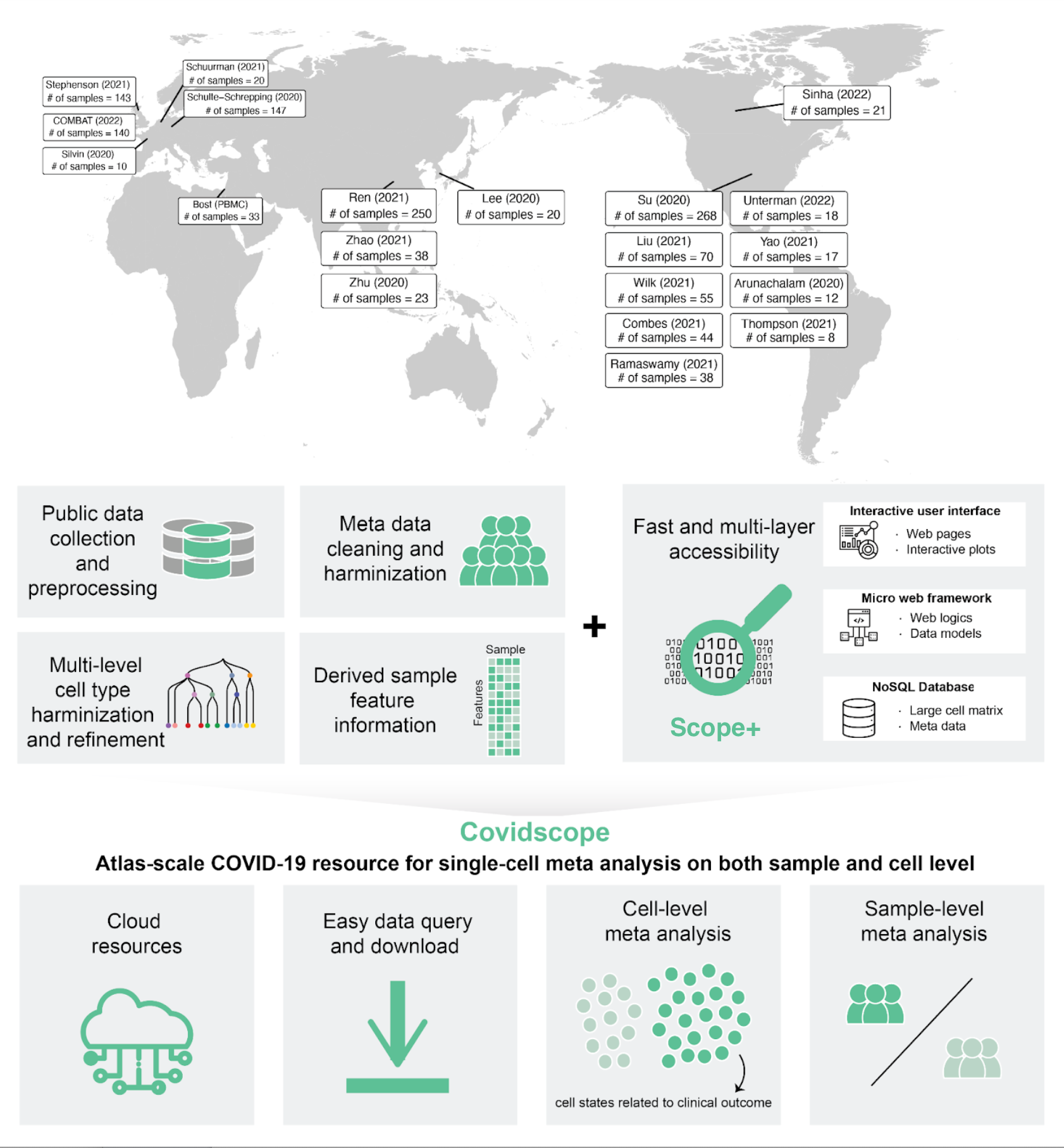
**

**Supplementary Figure 4:** **Schematic for large-scale COVID data integration, a portal in the cloud.**

Top panel shows the locations of the lead institutes of the 20 data sets. Bottom panel shows the schematic of the characteristics of the Covidscope web portal hosting the data sets and analytical results.


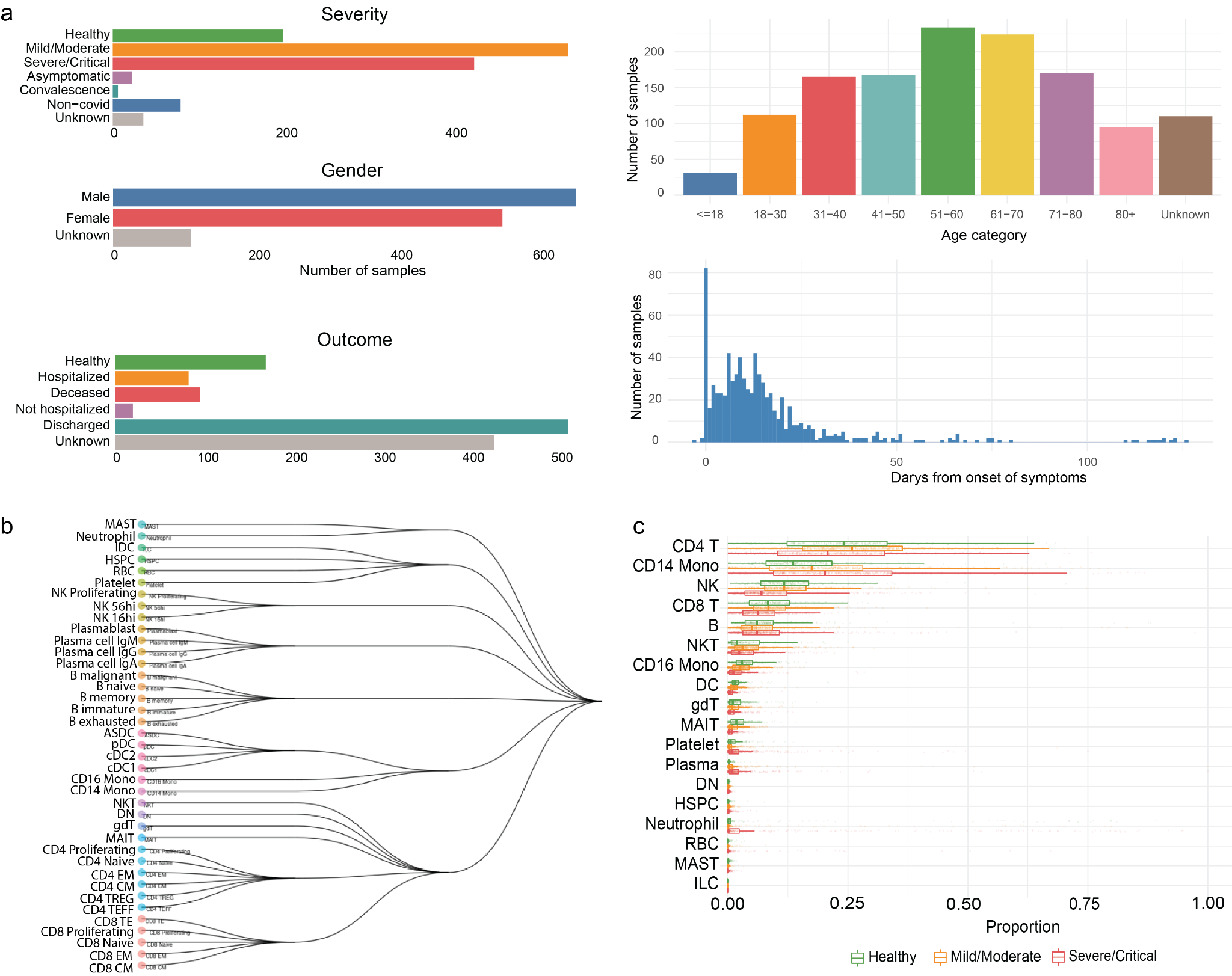


**Supplementary Figure 5: Data characteristics for the integrated COVID-19 data.**

**a** Shows the distribution of selected patient characteristics. **b** Uses the HOPACH clustering algorithm to construct the hierarchy of the cell types in the COVID-19 data atlas. **c** Shows the proportion of the cell types in patients of different severity.

**
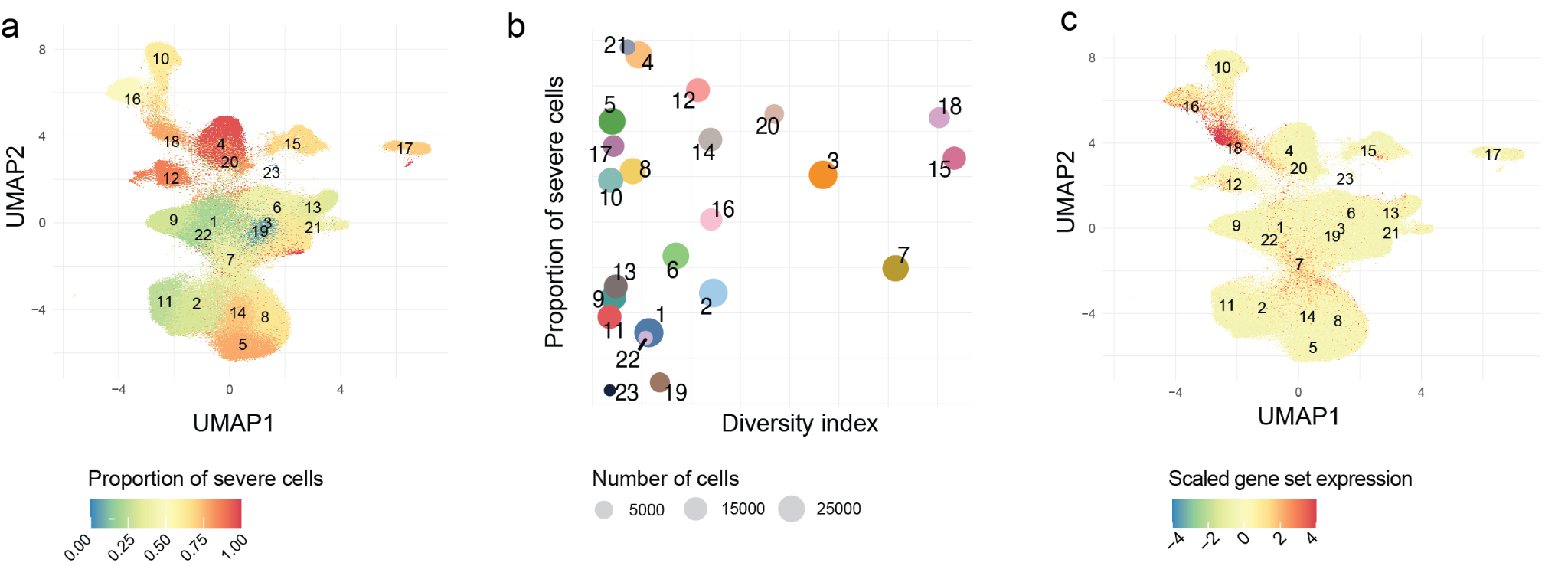
**

**Supplementary Figure 6: Case study on the CD14 monocyte cell population.**

**a** Shows the UMAP projection of the CD14 monocytes, with each derived cluster coloured by the proportion of severe cells in the cluster. **b** The diversity index quantifies the number of cells and number of data sets in each cluster, where a larger number represents more diversity. **c** The top 10 DE genes from cluster 18 and the remaining cluster were obtained. The scaled sum of gene expression from this gene set (IGLC3, IGLC2, IGHG2, IGHM, IGHG4, IGHG3, IGLC7, DEFA1B, FCER1A, IGHA2) were calculated for each cell and visualised.


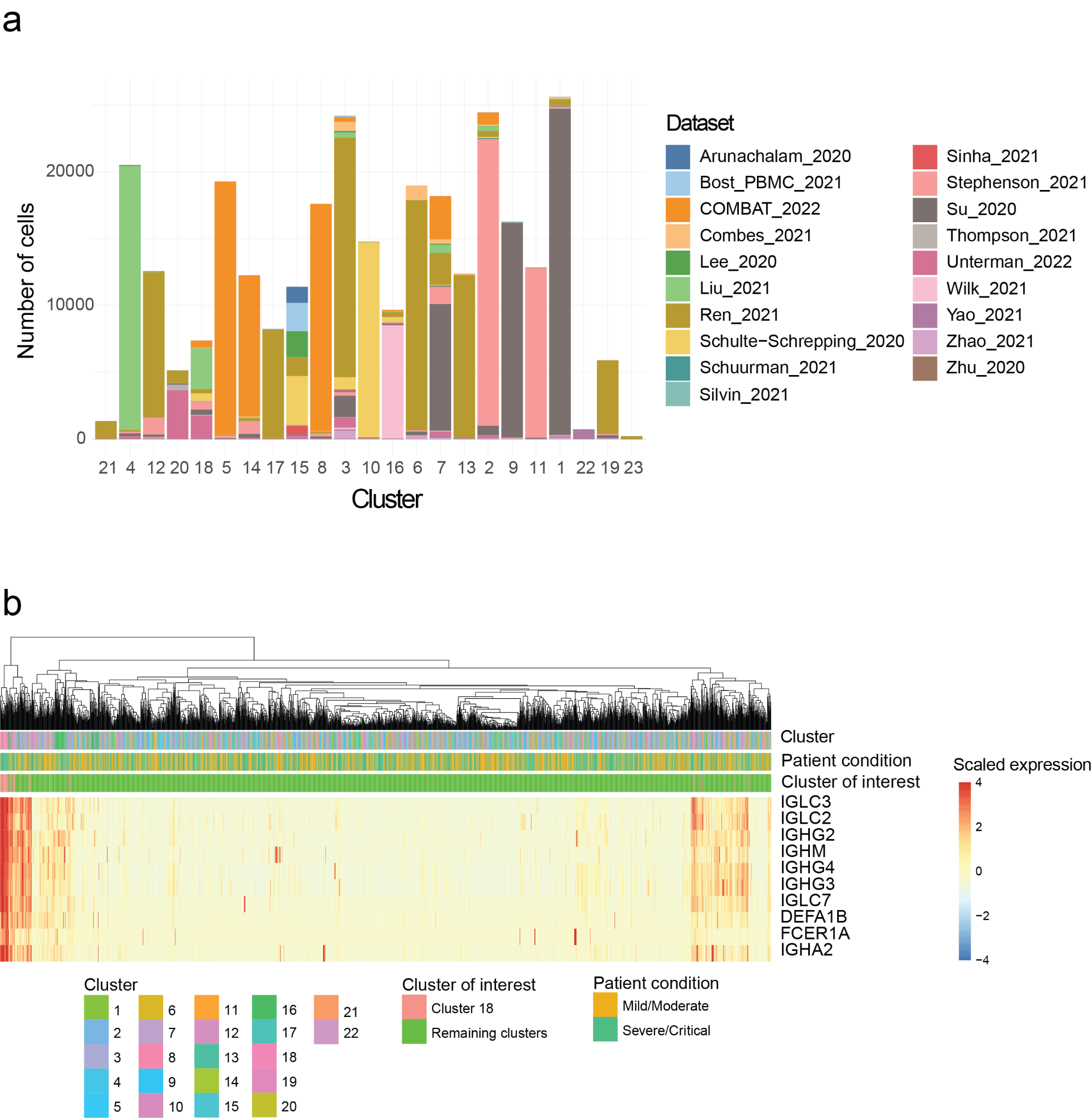


**Supplementary Figure 7. Differential expression analysis of the CD14 monocyte subclusters.**

**a** The barchart plots the number of cells belonging to each study in each cluster, with the clusters ordered by the greatest percentage of severe cells to the least percentage. **b** Shows the expression heatmap of the top 10 DE genes between cluster 18 and the remaining clusters.


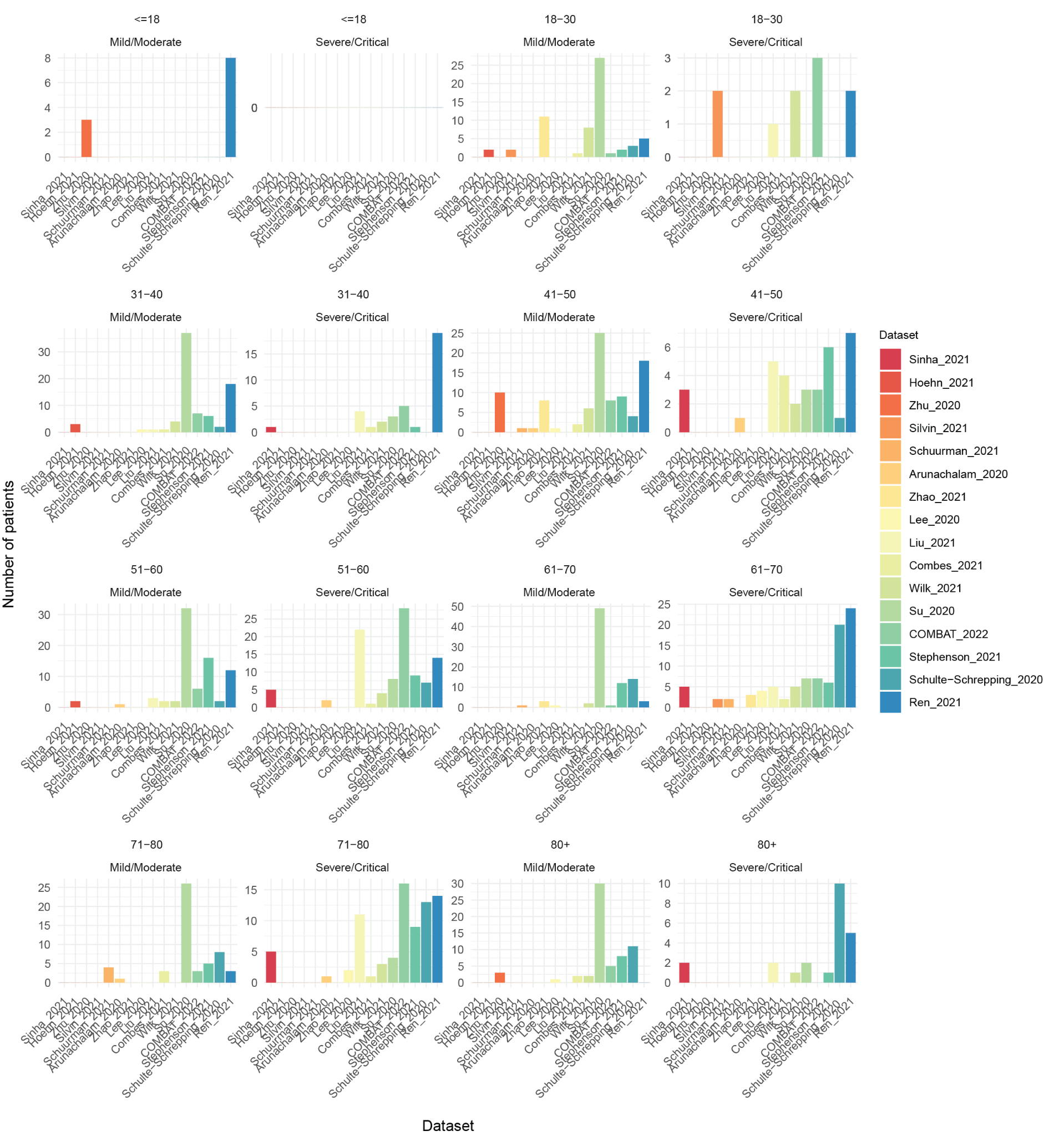


**Supplementary Figure 8. Age group distribution by data sets.**

Barplots show the age group distribution of the mild/moderate and severe/critical patients, coloured by data sets. Patients whose age is not reported in the original study were excluded. In the study, we referred to all mild/moderate patients as mild, and all severe/critical as severe.


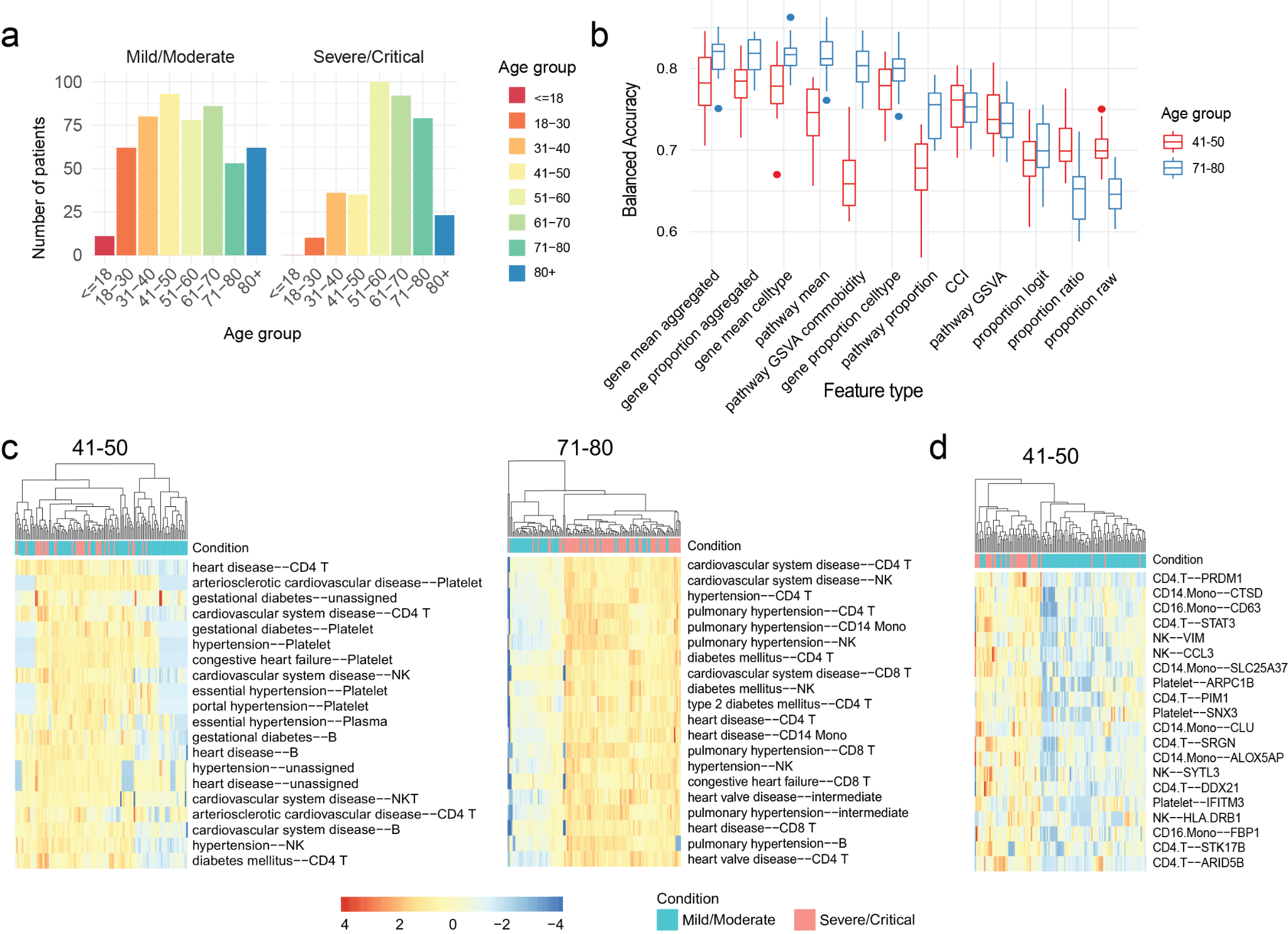


**Supplementary Figure 9: Case study of scFeatures on two distinct age groups of the COVID-19 patients.**

**a** Shows the age group distribution of mild/moderate and severe/critical patients in the database. In the study, we referred to all mild/moderate patients as mild, and all severe/critical as severe. We selected the 41-50 age group and the 71-80 age group and used scFeatures to generate molecular representation of individuals in each of the groups for further analysis. **b** Using mild and severe condition as the outcome, the boxplots show the classification accuracy using each feature type. Boxplots are coloured by age group. **c** Heatmaps show the values of the top 20 important features of the feature type “pathway GSVA comorbidity”, where the importance score was derived from classification model. Left shows the features on the 41-50 age group, right shows the features on the 71-80 age group. **d** Heatmap shows the values of the top 20 important features of the feature type “cell type gene mean” for the 41-50 age group.

**References:**

[Andreatta, Massimo, Fabrice P. A. David, Christian Iseli, Nicolas Guex, and Santiago J. Carmona. 2022. “SPICA: Swiss Portal for Immune Cell Analysis.” *Nucleic Acids Research* 50 (D1): D1109–14.](http://paperpile.com/b/oLcOhy/WAng)

[Arunachalam, Prabhu S., Florian Wimmers, Chris Ka Pun Mok, Ranawaka A. P. M. Perera, Madeleine Scott, Thomas Hagan, Natalia Sigal, et al. 2020. “Systems Biological Assessment of Immunity to Mild versus Severe COVID-19 Infection in Humans.” *Science* 369 (6508): 1210–20.](http://paperpile.com/b/oLcOhy/jVU8m)

[Bost, Pierre, Francesco De Sanctis, Stefania Canè, Stefano Ugel, Katia Donadello, Monica Castellucci, David Eyal, et al. 2021. “Deciphering the State of Immune Silence in Fatal COVID-19 Patients.” *Nature Communications* 12 (1): 1428.](http://paperpile.com/b/oLcOhy/jUu37)

[Cao, Junyue, Malte Spielmann, Xiaojie Qiu, Xingfan Huang, Daniel M. Ibrahim, Andrew J. Hill, Fan Zhang, et al. 2019. “The Single-Cell Transcriptional Landscape of Mammalian Organogenesis.” *Nature* 566 (7745): 496–502.](http://paperpile.com/b/oLcOhy/k75gA)

[Cao, Yue, Yingxin Lin, Ellis Patrick, Pengyi Yang, and Jean Yee Hwa Yang. 2022. “scFeatures: Multi-View Representations of Single-Cell and Spatial Data for Disease Outcome Prediction.” *Bioinformatics* , August, btac590.](http://paperpile.com/b/oLcOhy/BByOM)

[Chen, Sijie, Yanting Luo, Haoxiang Gao, Fanhong Li, Yixin Chen, Jiaqi Li, Renke You, et al. 2022. “hECA: The Cell-Centric Assembly of a Cell Atlas.” *iScience* 25 (5): 104318.](http://paperpile.com/b/oLcOhy/qUTN)

[Chen, Xinyue, Yin Huang, Liangfeng Huang, Ziliang Huang, Zhao-Zhe Hao, Lahong Xu, Nana Xu, et al. 2024. “A Brain Cell Atlas Integrating Single-Cell Transcriptomes across Human Brain Regions.” *Nature Medicine* 30 (9): 2679–91.](http://paperpile.com/b/oLcOhy/M3kb)

[Chen, Zhihang, Ziwei Luo, Di Zhang, Huiqin Li, Xuefei Liu, Kaiyu Zhu, Hongwan Zhang, et al. 2023. “TIGER: A Web Portal of Tumor Immunotherapy Gene Expression Resource.” *Genomics, Proteomics & Bioinformatics* 21 (2): 337–48.](http://paperpile.com/b/oLcOhy/XqxS)

[Combes, Alexis J., Tristan Courau, Nicholas F. Kuhn, Kenneth H. Hu, Arja Ray, William S. Chen, Nayvin W. Chew, et al. 2021. “Global Absence and Targeting of Protective Immune States in Severe COVID-19.” *Nature* 591 (7848): 124–30.](http://paperpile.com/b/oLcOhy/W9Ogo)

[COvid-19 Multi-omics Blood ATlas (COMBAT) Consortium, David J. Ahern, Zhichao Ai, Mark Ainsworth, Chris Allan, Alice Allcock, Azim Ansari, et al. 2021. “A Blood Atlas of COVID-19 Defines Hallmarks of Disease Severity and Specificity.” *bioRxiv*. medRxiv. https://doi.org/](http://paperpile.com/b/oLcOhy/a4mMe)[10.1101/2021.05.11.21256877](http://dx.doi.org/10.1101/2021.05.11.21256877)[.](http://paperpile.com/b/oLcOhy/a4mMe)

[CZI Single-Cell Biology Program, Shibla Abdulla, Brian Aevermann, Pedro Assis, Seve Badajoz, Sidney M. Bell, Emanuele Bezzi, et al. 2023. “CZ CELL×GENE Discover: A Single-Cell Data Platform for Scalable Exploration, Analysis and Modeling of Aggregated Data.” https://doi.org/](http://paperpile.com/b/oLcOhy/v6xZ)[10.1101/2023.10.30.563174](http://dx.doi.org/10.1101/2023.10.30.563174)[.](http://paperpile.com/b/oLcOhy/v6xZ)

[Gao, Xin, Fang Hong, Zhenyu Hu, Zilong Zhang, Yang Lei, Xiaoyun Li, and Tao Cheng. 2023. “ABC Portal: A Single-Cell Database and Web Server for Blood Cells.” *Nucleic Acids Research* 51 (D1): D792–804.](http://paperpile.com/b/oLcOhy/unck)

[Hossain, Calloway, and Lippa. 2019. “Visualization of Bioinformatics Data with Dash Bio.” *Proceedings of the Estonian Academy of Sciences. Biology, Ecology = Eesti Teaduste Akadeemia Toimetised. Bioloogia, Okoloogia*.](http://paperpile.com/b/oLcOhy/atPMD) <https://conference.scipy.org/proceedings/scipy2019/pdfs/shammamah_hossain.pdf>[.](http://paperpile.com/b/oLcOhy/atPMD)

[HuBMAP Consortium. 2019. “The Human Body at Cellular Resolution: The NIH Human Biomolecular Atlas Program.” *Nature* 574 (7777): 187–92.](http://paperpile.com/b/oLcOhy/4J3I)

[Inc., Plotly Technologies. 2015. “Collaborative Data Science.” Montreal, QC: Plotly Technologies Inc. 2015.](http://paperpile.com/b/oLcOhy/mvoRp) <https://plot.ly>[.](http://paperpile.com/b/oLcOhy/mvoRp)

[Jin, Kang, Eric E. Bardes, Alexis Mitelpunkt, Jake Y. Wang, Surbhi Bhatnagar, Soma Sengupta, Daniel Pomeranz Krummel, Marc E. Rothenberg, and Bruce J. Aronow. 2021. “An Interactive Single Cell Web Portal Identifies Gene and Cell Networks in COVID-19 Host Responses.” *iScience* 24 (10): 103115.](http://paperpile.com/b/oLcOhy/RqZi)

[Lee, Jeong Seok, Seongwan Park, Hye Won Jeong, Jin Young Ahn, Seong Jin Choi, Hoyoung Lee, Baekgyu Choi, et al. 2020. “Immunophenotyping of COVID-19 and Influenza Highlights the Role of Type I Interferons in Development of Severe COVID-19.” *Science Immunology* 5 (49). https://doi.org/](http://paperpile.com/b/oLcOhy/eoSuj)[10.1126/sciimmunol.abd1554](http://dx.doi.org/10.1126/sciimmunol.abd1554)[.](http://paperpile.com/b/oLcOhy/eoSuj)

[Li, Mengwei, Xiaomeng Zhang, Kok Siong Ang, Jingjing Ling, Raman Sethi, Nicole Yee Shin Lee, Florent Ginhoux, and Jinmiao Chen. 2022. “DISCO: A Database of Deeply Integrated Human Single-Cell Omics Data.” *Nucleic Acids Research* 50 (D1): D596–602.](http://paperpile.com/b/oLcOhy/vEKG)

[Lin, Dongdong, Yirui Chen, Soumya Negi, Derrick Cheng, Zhengyu Ouyang, David Sexton, Kejie Li, and Baohong Zhang. 2021. “CellDepot: A Unified Repository for scRNA-Seq Data and Visual Exploration.” https://doi.org/](http://paperpile.com/b/oLcOhy/G7hv)[10.1101/2021.09.30.462602](http://dx.doi.org/10.1101/2021.09.30.462602)[.](http://paperpile.com/b/oLcOhy/G7hv)

[Lin, Yingxin, Yue Cao, Hani Jieun Kim, Agus Salim, Terence P. Speed, David M. Lin, Pengyi Yang, and Jean Yee Hwa Yang. 2020. “scClassify: Sample Size Estimation and Multiscale Classification of Cells Using Single and Multiple Reference.” *Molecular Systems Biology* 16 (6): e9389.](http://paperpile.com/b/oLcOhy/yzKXA)

[Lin, Yingxin, Yue Cao, Elijah Willie, Ellis Patrick, and Jean Y. H. Yang. 2023. “Atlas-Scale Single-Cell Multi-Sample Multi-Condition Data Integration Using scMerge2.” *Nature Communications* 14 (1): 4272.](http://paperpile.com/b/oLcOhy/INMRF)

[Liu, Can, Andrew J. Martins, William W. Lau, Nicholas Rachmaninoff, Jinguo Chen, Luisa Imberti, Darius Mostaghimi, et al. 2021. “Time-Resolved Systems Immunology Reveals a Late Juncture Linked to Fatal COVID-19.” *Cell* 184 (7): 1836–57.e22.](http://paperpile.com/b/oLcOhy/qHC88)

[Ma, Wei Feng, Adam W. Turner, Christina Gancayco, Doris Wong, Yipei Song, Jose Verdezoto Mosquera, Gaëlle Auguste, et al. 2022. “PlaqView 2.0: A Comprehensive Web Portal for Cardiovascular Single-Cell Genomics.” *Frontiers in Cardiovascular Medicine* 9 (August):969421.](http://paperpile.com/b/oLcOhy/r7q6)

[Nie, Hu, Peilu Lin, Yu Zhang, Yihong Wan, Jiesheng Li, Chengqian Yin, and Lei Zhang. 2023. “Single-Cell Meta-Analysis of Inflammatory Bowel Disease with scIBD.” *Nature Computational Science* 3 (6): 522–31.](http://paperpile.com/b/oLcOhy/kd0g)

[Nieto, Paula, Marc Elosua-Bayes, Juan L. Trincado, Domenica Marchese, Ramon Massoni-Badosa, Maria Salvany, Ana Henriques, et al. 2021. “A Single-Cell Tumor Immune Atlas for Precision Oncology.” *Genome Research* 31 (10): 1913–26.](http://paperpile.com/b/oLcOhy/EJWR)

[Pan, Lu, Paolo Parini, Roman Tremmel, Joseph Loscalzo, Volker M. Lauschke, Bradley A. Maron, Paola Paci, et al. 2024. “Single Cell Atlas: A Single-Cell Multi-Omics Human Cell Encyclopedia.” *Genome Biology* 25 (1): 104.](http://paperpile.com/b/oLcOhy/iZVz)

[Qi, Changlu, Chao Wang, Lingling Zhao, Zijun Zhu, Ping Wang, Sainan Zhang, Liang Cheng, and Xue Zhang. 2022. “SCovid: Single-Cell Atlases for Exposing Molecular Characteristics of COVID-19 across 10 Human Tissues.” *Nucleic Acids Research* 50 (D1): D867–74.](http://paperpile.com/b/oLcOhy/h14r)

[Ramaswamy, Anjali, Nina N. Brodsky, Tomokazu S. Sumida, Michela Comi, Hiromitsu Asashima, Kenneth B. Hoehn, Ningshan Li, et al. 2021. “Immune Dysregulation and Autoreactivity Correlate with Disease Severity in SARS-CoV-2-Associated Multisystem Inflammatory Syndrome in Children.” *Immunity* 54 (5): 1083–95.e7.](http://paperpile.com/b/oLcOhy/Ms85l)

[Regev, Aviv, Sarah A. Teichmann, Eric S. Lander, Ido Amit, Christophe Benoist, Ewan Birney, Bernd Bodenmiller, et al. 2017. “The Human Cell Atlas.” *eLife* 6 (December). https://doi.org/](http://paperpile.com/b/oLcOhy/zIDr)[10.7554/eLife.27041](http://dx.doi.org/10.7554/eLife.27041)[.](http://paperpile.com/b/oLcOhy/zIDr)

[Ren, Xianwen, Wen Wen, Xiaoying Fan, Wenhong Hou, Bin Su, Pengfei Cai, Jiesheng Li, et al. 2021. “COVID-19 Immune Features Revealed by a Large-Scale Single-Cell Transcriptome Atlas.” *Cell* 184 (23): 5838.](http://paperpile.com/b/oLcOhy/bg3mM)

[Rozenblatt-Rosen, Orit, Aviv Regev, Philipp Oberdoerffer, Tal Nawy, Anna Hupalowska, Jennifer E. Rood, Orr Ashenberg, et al. 2020. “The Human Tumor Atlas Network: Charting Tumor Transitions across Space and Time at Single-Cell Resolution.” *Cell* 181 (2): 236–49.](http://paperpile.com/b/oLcOhy/YttH)

[Schulte-Schrepping, Jonas, Nico Reusch, Daniela Paclik, Kevin Baßler, Stephan Schlickeiser, Bowen Zhang, Benjamin Krämer, et al. 2020. “Severe COVID-19 Is Marked by a Dysregulated Myeloid Cell Compartment.” *Cell* 182 (6): 1419–40.e23.](http://paperpile.com/b/oLcOhy/ItQbY)

[Schuurman, Alex R., Tom D. Y. Reijnders, Anno Saris, Ivan Ramirez Moral, Michiel Schinkel, Justin de Brabander, Christine van Linge, et al. 2021. “Integrated Single-Cell Analysis Unveils Diverging Immune Features of COVID-19, Influenza, and Other Community-Acquired Pneumonia.” *eLife* 10 (August). https://doi.org/](http://paperpile.com/b/oLcOhy/Jqn7F)[10.7554/eLife.69661](http://dx.doi.org/10.7554/eLife.69661)[.](http://paperpile.com/b/oLcOhy/Jqn7F)

[Shen, Zhuoqiao, Minghao Fang, Wujianan Sun, Meifang Tang, Nianping Liu, Lin Zhu, Qian Liu, et al. 2022. “A Transcriptome Atlas and Interactive Analysis Platform for Autoimmune Disease.” *Database: The Journal of Biological Databases and Curation* 2022 (June). https://doi.org/](http://paperpile.com/b/oLcOhy/6li8)[10.1093/database/baac050](http://dx.doi.org/10.1093/database/baac050)[.](http://paperpile.com/b/oLcOhy/6li8)

[Silvin, Aymeric, Nicolas Chapuis, Garett Dunsmore, Anne-Gaëlle Goubet, Agathe Dubuisson, Lisa Derosa, Carole Almire, et al. 2020. “Elevated Calprotectin and Abnormal Myeloid Cell Subsets Discriminate Severe from Mild COVID-19.” *Cell* 182 (6): 1401–18.e18.](http://paperpile.com/b/oLcOhy/3HL9A)

[Sinha, Sarthak, Nicole L. Rosin, Rohit Arora, Elodie Labit, Arzina Jaffer, Leslie Cao, Raquel Farias, et al. 2022. “Dexamethasone Modulates Immature Neutrophils and Interferon Programming in Severe COVID-19.” *Nature Medicine* 28 (1): 201–11.](http://paperpile.com/b/oLcOhy/WPR3L)

[Stephenson, Emily, Gary Reynolds, Rachel A. Botting, Fernando J. Calero-Nieto, Michael D. Morgan, Zewen Kelvin Tuong, Karsten Bach, et al. 2021. “Single-Cell Multi-Omics Analysis of the Immune Response in COVID-19.” *Nature Medicine* 27 (5): 904–16.](http://paperpile.com/b/oLcOhy/hv3Wh)

[Sungnak, Waradon, Ni Huang, Christophe Bécavin, Marijn Berg, Rachel Queen, Monika Litvinukova, Carlos Talavera-López, et al. 2020. “SARS-CoV-2 Entry Factors Are Highly Expressed in Nasal Epithelial Cells Together with Innate Immune Genes.” *Nature Medicine* 26 (5): 681–87.](http://paperpile.com/b/oLcOhy/Uiz1)

[Su, Yapeng, Daniel Chen, Dan Yuan, Christopher Lausted, Jongchan Choi, Chengzhen L. Dai, Valentin Voillet, et al. 2020. “Multi-Omics Resolves a Sharp Disease-State Shift between Mild and Moderate COVID-19.” *Cell* 183 (6): 1479–95.e20.](http://paperpile.com/b/oLcOhy/6guox)

[Tabula Sapiens Consortium*, Robert C. Jones, Jim Karkanias, Mark A. Krasnow, Angela Oliveira Pisco, Stephen R. Quake, Julia Salzman, et al. 2022. “The Tabula Sapiens: A Multiple-Organ, Single-Cell Transcriptomic Atlas of Humans.” *Science* 376 (6594): eabl4896.](http://paperpile.com/b/oLcOhy/KHsa)

[Thompson, Elizabeth A., Katherine Cascino, Alvaro A. Ordonez, Weiqiang Zhou, Ajay Vaghasia, Anne Hamacher-Brady, Nathan R. Brady, et al. 2021. “Metabolic Programs Define Dysfunctional Immune Responses in Severe COVID-19 Patients.” *Cell Reports* 34 (11): 108863.](http://paperpile.com/b/oLcOhy/4lqWX)

[Tian, Yuan, Lindsay N. Carpp, Helen E. R. Miller, Michael Zager, Evan W. Newell, and Raphael Gottardo. 2022. “Single-Cell Immunology of SARS-CoV-2 Infection.” *Nature Biotechnology* 40 (1): 30–41.](http://paperpile.com/b/oLcOhy/uDto)

[Unterman, Avraham, Tomokazu S. Sumida, Nima Nouri, Xiting Yan, Amy Y. Zhao, Victor Gasque, Jonas C. Schupp, et al. 2022. “Single-Cell Multi-Omics Reveals Dyssynchrony of the Innate and Adaptive Immune System in Progressive COVID-19.” *Nature Communications* 13 (1): 440.](http://paperpile.com/b/oLcOhy/DRwrW)

[Wilk, Aaron J., Madeline J. Lee, Bei Wei, Benjamin Parks, Ruoxi Pi, Giovanny J. Martínez-Colón, Thanmayi Ranganath, et al. 2021. “Multi-Omic Profiling Reveals Widespread Dysregulation of Innate Immunity and Hematopoiesis in COVID-19.” *The Journal of Experimental Medicine* 218 (8). https://doi.org/](http://paperpile.com/b/oLcOhy/ieply)[10.1084/jem.20210582](http://dx.doi.org/10.1084/jem.20210582)[.](http://paperpile.com/b/oLcOhy/ieply)

[Yang, Xiaoxiao, Yang Tong, Gerui Liu, Jiapei Yuan, and Yang Yang. 2022. “scAPAatlas: An Atlas of Alternative Polyadenylation across Cell Types in Human and Mouse.” *Nucleic Acids Research* 50 (D1): D356–64.](http://paperpile.com/b/oLcOhy/wltW)

[Yao, Changfu, Stephanie A. Bora, Tanyalak Parimon, Tanzira Zaman, Oren A. Friedman, Joseph A. Palatinus, Nirmala S. Surapaneni, et al. 2021. “Cell-Type-Specific Immune Dysregulation in Severely Ill COVID-19 Patients.” *Cell Reports* 34 (13): 108943.](http://paperpile.com/b/oLcOhy/xV0wA)

[Yu, Guangchuang, Li-Gen Wang, Guang-Rong Yan, and Qing-Yu He. 2015. “DOSE: An R/Bioconductor Package for Disease Ontology Semantic and Enrichment Analysis.” *Bioinformatics*  31 (4): 608–9.](http://paperpile.com/b/oLcOhy/1JuGV)

[Yu, Lijia, Yue Cao, Jean Y. H. Yang, and Pengyi Yang. 2022. “Benchmarking Clustering Algorithms on Estimating the Number of Cell Types from Single-Cell RNA-Sequencing Data.” *Genome Biology* 23 (1): 49.](http://paperpile.com/b/oLcOhy/k0n6M)

[Zeng, Jingyao, Yadong Zhang, Yunfei Shang, Jialin Mai, Shuo Shi, Mingming Lu, Congfan Bu, et al. 2022. “CancerSCEM: A Database of Single-Cell Expression Map across Various Human Cancers.” *Nucleic Acids Research* 50 (D1): D1147–55.](http://paperpile.com/b/oLcOhy/DNnW)

[Zhao, Xiang-Na, Yue You, Xiao-Ming Cui, Hui-Xia Gao, Guo-Lin Wang, Sheng-Bo Zhang, Lin Yao, et al. 2021. “Single-Cell Immune Profiling Reveals Distinct Immune Response in Asymptomatic COVID-19 Patients.” *Signal Transduction and Targeted Therapy* 6 (1): 342.](http://paperpile.com/b/oLcOhy/2ufUk)

[Zhou, Yadi, Jielin Xu, Yuan Hou, Lynn Bekris, James B. Leverenz, Andrew A. Pieper, Jeffrey Cummings, and Feixiong Cheng. 2022. “The Alzheimer’s Cell Atlas (TACA): A Single-Cell Molecular Map for Translational Therapeutics Accelerator in Alzheimer's Disease.” *Alzheimer’s & Dementia: The Journal of the Alzheimer's Association* 8 (1): e12350.](http://paperpile.com/b/oLcOhy/6KZ7)

[Zhu, Linnan, Penghui Yang, Yingze Zhao, Zhenkun Zhuang, Zhifeng Wang, Rui Song, Jie Zhang, et al. 2020. “Single-Cell Sequencing of Peripheral Mononuclear Cells Reveals Distinct Immune Response Landscapes of COVID-19 and Influenza Patients.” *Immunity* 53 (3): 685–96.e3.](http://paperpile.com/b/oLcOhy/0Cf9j)
